# Flurofamide Prevention and Treatment of *Ureaplasma*-Induced Hyperammonemia

**DOI:** 10.1128/spectrum.01927-22

**Published:** 2022-08-22

**Authors:** Derek Fleming, Robin Patel

**Affiliations:** a Division of Clinical Microbiology, Department of Laboratory Medicine and Pathology, Mayo Clinic, Rochester, Minnesota, USA; b Division of Public Health, Infectious Diseases and Occupational Medicine, Mayo Clinic, Rochester, Minnesota, USA; Quest Diagnostics

**Keywords:** *Ureaplasma*, hyperammonemia, lung transplantation

## Abstract

Hyperammonemia (HA) syndrome caused by respiratory infection with ammonia (NH_3_)-producing *Ureaplasma* species occurs in 4% of lung transplant recipients (LTRs) and is associated with high mortality. Although *Ureaplasma*-targeted antibiotic intervention is effective, the threat of antibiotic resistance development and pre-existing resistance make an alternative to antibiotics desirable. Considering that the underlying pathology of *Ureaplasma*-induced hyperammonemia (UIHA) is dependent upon ureaplasmal urease converting urea to NH_3_, urease inhibition could represent a targeted treatment approach. Here, the ability of the urease inhibitor, flurofamide, to prevent and treat UIHA was investigated. To confirm that flurofamide is broadly active against *Ureaplasma* respiratory isolates, the minimum urease inhibitory concentration against 4 isolates of Ureaplasma parvum and 5 isolates of Ureaplasma urealyticum was first determined *in vitro*. NH_3_ production by all isolates was inhibited by ≤2 μM flurofamide. To test the ability of flurofamide to prevent and treat UIHA, a mouse model of *Ureaplasma* respiratory infection was utilized. When animals were administered 6 mg/kg flurofamide via intraperitoneal injection 1 h prior to infection with U. parvum, flurofamide-administered animals exhibited significantly lower blood NH_3_ levels than did non-prophylaxed animals (10.9 ± 4.0 μmol/L compared to 26.5 ± 17.7 μmol/L; *P* = 0.0146) 24 h post-treatment. When U. parvum-infected hyperammonemic mice were treated with 6 mg/kg flurofamide, treated animals had significantly greater decreases in blood-NH_3_ levels 6 h post-treatment than did untreated mice (56.4 ± 17.1% compared to 9.1 ± 33.5% reduction; *P* = 0.0152). Together, these results indicate that flurofamide is a promising non-antibiotic treatment for UIHA in LTRs.

**IMPORTANCE** Ureaplasma-associated hyperammonemia syndrome occurs in 4% of lung transplant recipients and has historically been almost universally fatal. While Ureaplasma-targeted antibiotics have been shown to be protective, the possibility of underlying resistance and resistance selection render non-antibiotic interventions an interesting approach.

## INTRODUCTION

Hyperammonemia (HA) syndrome resulting from infection of the respiratory tract by Ureaplasma species has historically occurred in approximately 4% of early-post-operative lung transplant recipients (LTRs), and is associated with high mortality (1–5). Ureaplasmal urease splits urea into ammonia (NH_3_) and CO_2_, generating an NH_3_ gradient across the bacterial membrane that powers a unique ATP synthase (6, 7). This drives 95% of ureaplasmal ATP synthesis, making urea a requirement for growth of the organisms (8). LTRs who become colonized with the human-associated Ureaplasma species, U. urealyticum and U. parvum, are particularly vulnerable to bacterial overproduction of NH_3_ to such a degree that it overwhelms host detoxification capacity, resulting in cerebral edema and often death (9–13). Although the mechanisms that make LTRs exceptionally vulnerable to this clinical manifestation are not completely understood, the heavily immunocompromised status of LTRs, coupled with infection site advantages, such as ischemic tissue resulting from incomplete lung revascularization, and increased urea availability due to uremia resulting from acute renal failure (14), are likely contributing factors. Although Ureaplasma-directed antibiotic therapy can be curative and preventative in these patients, greatly reducing mortality (15, 16), antibiotic use can lead to selection of resistance, resulting in relapse and treatment failure (1). In addition, underlying resistance at initial diagnosis, which is not typically quickly ascertained, may compromise therapy, therefore, non-antibiotic treatment options are of interest. Considering that pathogenesis is due to the action of ureaplasmal ureases, it was hypothesized that pharmacological urease inhibition would prevent and treat Ureaplasma-induced hyperammonemia (UIHA).

Flurofamide (*N*-[diami-nophosphinyl]-4-fluorobenzamide), a derivative of phosphoric triamide (17), is a urease inhibitor that has been shown to limit growth of Ureaplasma species *in vitro* and *in vivo* (18, 19), and to protect mice from mortality caused by intravenous injection of Ureaplasma cells or Ureaplasma sonicate (20). In this work, the ability of flurofamide to protect against UIHA when administered either before infection, or after the onset of hyperammonemia, was explored.

## RESULTS

### All tested *Ureaplasma* isolates were inhibited by flurofamide *in vitro*.

For all U. parvum and U. urealyticum isolates tested, flurofamide inhibited NH_3_ production at minimum urease inhibitory concentrations of no greater than 2 μM (Table 1), confirming that flurofamide is a broad-spectrum inhibitor of ureaplasmal urease.

**TABLE 1:** Minimum urease inhibitory concentrations of flurofamide against Ureaplasma isolates

Species	Isolate #	Source	Flurofamide minimum urease inhibitory (μM)
*U. parvum*	ATCC-27815	Urethritis	0.5
*U. parvum*	IDRL-11887	Bronchoalveolar Lavage Fluid	2
*U. parvum*	IDRL-10774	Bronchoalveolar Lavage Fluid	2
*U. parvum*	IDRL-11264	Sputum	0.5
*U. urealyticum*	ATCC-27816	Urethritis	2
*U. urealyticum*	IDRL-10612	Bronchoalveolar Lavage Fluid	2
*U. urealyticum*	IDRL-10763	Bronchial Washings	0.06
*U. urealyticum*	IDRL-11235	Tracheal Secretions	2
*U. urealyticum*	IDRL-12698	Bronchoalveolar Lavage Fluid	2

### Flurofamide prevented *Ureaplasma*-induced hyperammonemia in mice.

**FIG 1:**
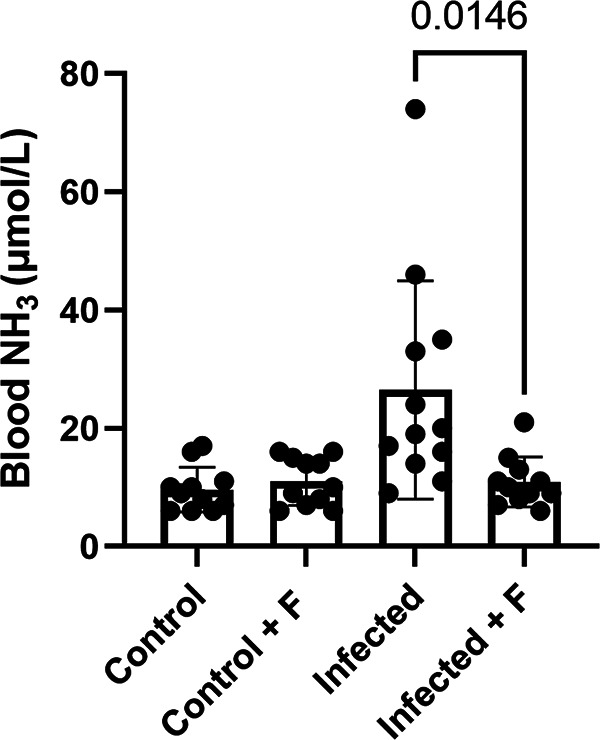
Flurofamide prevents Ureaplasma-induced hyperammonemia. Mice were administered 6 mg/kg IP flurofamide 1 h prior to infection with U. parvum IDRL-10774. After 24 h, blood NH_3_ was measured. Control groups were saline vehicle (control) with and without flurofamide (+ F) and infected without flurofamide. N = 12 for infected, N = 11 for infected with flurofamide, and N = 11 for each control group. Significance between groups was determined via two-tailed unpaired t-tests.

When mice were administered 6 mg/kg flurofamide 1 h prior to intratracheal (IT) and intraperitoneal (IP) infection with a U. parvum respiratory isolate, IDRL-10774, resultant 24-h-postinfection blood NH_3_ levels were lower than in infected mice not administered flurofamide. Specifically, flurofamide-administered animals exhibited an average blood NH_3_ level of 10.9 ± 4.03 μmol/L, compared to 26.5 ± 17.7 μmol/L for non-prophylaxed animals (*P* = 0.0146) (Fig. 1). Uninfected control animals (saline vehicle), with and without flurofamide administration exhibited blood NH_3_ levels of 9.64 ± 3.65 and 11.00 ± 3.86 μmol/L, respectively. Ureaplasma loads for treated and untreated groups, as determined by quantititave PCR (qPCR) were not significantly different for lungs (2.4 ± 2.8 × 10^4^ versus 4.1 ± 5.9 × 10^4^ total copies, respectively; *P* = 0.4152) or blood (8.1 ± 22.6 × 10^2^ versus 3.0 ± 4.5 × 10^2^ copies/mL, respectively; *P* = 0.4706), indicating that reductions in NH_3_ production were a result of urease inhibition, not bacterial growth inhibition. qPCR copy counts for most blood samples were below the limit of detection for the assay (<5 × 10^2^ copies/mL), even for hyperammonemic mice, indicating that systemic infection is not necessary to achieve elevated blood NH_3_ in this model.

### Flurofamide treatment resolved *Ureaplasma*-induced hyperammonemia in mice.

**FIG 2:**
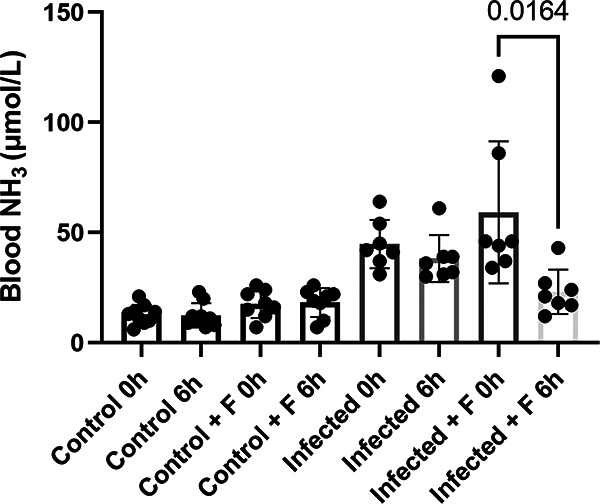
Flurofamide treatment lowers blood NH_3_ levels in Ureaplasma-induced hyperammonemia. Mice were infected with U. parvum IDRL-10774. After 24 h, blood NH_3_ was measured (0 h) and infected animals with >30 μmol/L NH_3_ were divided into treatment and no treatment groups. Mice in the treatment group were administered 6 mg/kg IP flurofamide, and after 6 h of treatment, blood NH_3_ levels were measured (6 h). Control groups were saline vehicle (control) with and without flurofamide (+ F) and infected without flurofamide. N = 7 for each infected group, and N = 9 for each control group. Significance between groups was determined via two-tailed unpaired t-tests.

When hyperammonemic mice were treated with 6 mg/kg flurofamide 24 h after infection with a U. parvum respiratory isolate IDRL-10774, treated animals exhibited significantly reduced blood NH_3_ levels compared to untreated mice (Fig. 2). Only animals with blood NH_3_ levels greater than 30 μmol/L post infection were included in the treated and untreated groups (divided equally between the groups). Flurofamide treated animals had 56.4 ± 17.2% reductions in blood NH_3_ levels 6 h post-treatment, compared to only 9.1 ± 33.5% reductions for untreated animals. Uninfected control animals (saline vehicle), with and without flurofamide treatment, exhibited 1.1 ± 23.2% average increases and 7.3 ± 23.2% average decreases, respectively (*P* = 0.0152). As with prophylaxis, no significant differences in bacterial loads between treated and untreated groups were detected in the lungs (1.1 ± 1.2 × 10^2^ versus 1.1 ± 2.0 × 10^3^ total copies, respectively; *P* = 0.3858) or blood (all below limit of detection).

## DISCUSSION

UIHA is a serious complication of lung transplantation than can be prevented and treated with Ureaplasma-targeted antibiotic therapy. However, the threat of antibiotic resistance, and the possibility of persistent pathophysiology associated with incompletely killed Ureaplasma populations and/or residually active ureases, threatens clinical efficacy. Antibiotic resistance in Ureaplasma species can be pre-existing; resistance testing for Ureaplasma can be challenging to perform and slow, rendering antibiotic susceptibility data unlikely to be quickly available to treating clinicians (15, 21). Furthermore, resistance may be selected for with antibiotic therapy itself. Also, non-antibiotic approaches are useful in avoiding microbiome disturbances and selection of antibiotic resistance in commensal microbiota. For these reasons, exploring non-antibiotic alternative therapies that minimize undesirable collateral outcomes is of interest. Considering that NH_3_ production by ureaplasmal ureases is the underlying mechanism of UIHA, urease inhibition would provide an alternative, more-targeted approach than antibiotics. In this study, the ability of flurofamide, a potent urease inhibitor, to prevent and treat UIHA in an experimental mouse model was tested.

To confirm broad-spectrum urease inhibition in U. parvum and U. urealyticum, an *in vitro* urease inhibition assay was performed on seven clinical respiratory Ureaplasma isolates and 2 commercially available urethritis isolates. It was found that NH_3_ production was inhibited at concentrations no greater than 2 μM for all isolates, indicating that flurofamide is broadly active against urease from infectious Ureaplasma.

Ideally, flurofamide could be used to both prevent UIHA and treat established UIHA in patients undergoing lung transplantation or other solid organ transplantation. Thus, a murine model of Ureaplasma pneumonia was utilized to test flurofamide prophylactically and therapeutically. A single dose of 6 mg/kg flurfamide either 1 h before infection with U. parvum, or after UIHA had been established (24 h post infection with U. parvum), resulted in lower resultant blood NH_3_ levels compared to infected, untreated mice and uninfected mice with and without flurofamide. Together, these results show that flurofamide is a promising non-antibiotic treatment option for the prevention, and resolution, of UIHA.

This study has several limitations. First, considering the previous studies of flurofamide against Ureaplasma species (18, 19), with the *in vitro* arm of this work we sought to confirm activity against respiratory isolates from LTRs with UIHA. Given the novelty of our understanding that Ureaplasma infection is the causative agent of this infrequent condition, a limited number of such isolates were available. Ideally, more isolates would be tested to demonstrate broad inhibitory ability. Further, while the ability of flurofamide to inhibit urease from various clinical Ureaplasma isolates was shown *in vitro*, only a single clinical respiratory isolate was tested in the mouse model. In the interest of limiting animal work, U. parvum IDRL-10774 was used as a representative isolate. This isolate was chosen due to its *in vitro* minimum urease inhibitory concentration being among the highest of all isolates tested, making it a “worse-case scenario” causative agent. Next, only a single flurofamide concentration, given in a single dose, was studied. It is possible that higher doses and/or doses given repetitively would improve efficacy; they should be studied in more detail in the future. Lastly, it should be noted that the animals in this study experienced mild HA, and thus the ability of flurofamide to treat more severe cases will require further investigation. Given the established link of uremia to increased ureaplasmal NH_3_ production (14), an acute kidney injury model could be used in place of the dietary uremia model in future studies.

In conclusion, flurofamide is a promising non-antibiotic approach to specifically target ureaplasmal ureases for prevention and treatment of UIHA in LTRs.

## MATERIALS AND METHODS

### Study isolates.

Three respiratory isolates of U. parvum, 4 respiratory isolates of U. urealyticum, and 1 commercially available urogenital isolate of each species (ATCC) were studied. Patient respiratory isolates were acquired from the Clinical Microbiology Laboratory at Mayo Clinic, Rochester. All respiratory isolates were from patients with UIHA. Isolation was performed on Ureaplasma-specific A8 agar (Hardy Diagnostics), and species identification was performed via PCR, with differences under the probes resulting in different melting temperatures for the 2 species.

Isolates were grown to 10^8^ to 10^9^ copies/mL using a previously described Ureaplasma bioreactor (22). 500 μL aliquots in U9 broth (Hardy Diagnostics) buffered with 100 mM 2-ethanesulfonic acid (MES) at pH 6.0 were frozen at −80°C until use.

### *In vitro* minimum urease inhibitory concentration determination.

Ureaplasma aliquots were pelleted at 15,000 × G for 20 min, resuspended in unbuffered 10B broth (Remel), and diluted to 10^6^ copies/mL. For each isolate, 3 rows (replicates) of the same 96-well plate were filled with 100 μL of fresh 10B containing between 0 and 32 μM flurofamide (Tocris Bioscience). To those wells, 10 μL of Ureaplasma suspension was added, and the plates incubated at 37°C, without shaking, until control wells (containing no flurofamide) turned from yellow to fushcia (phenol red indicator). Inoculations were performed at night and observed through the next day to allow for monitoring of color change, which usually occurred between 14 and 18 h. At that time, the lowest concentration that failed to turn fushcia upon visual inspection was recorded as the minimum urease inhibitory concentration. If disagreement occurred between repeats, the highest concentration needed to inhibit color change was recorded, provided the difference was no more than one doubling dilution.

### *In vivo* flurofamide prophylaxis.

U. parvum IDRL-10774 aliquots were pelleted at 15,000 × G for 20 min at 4°C and resuspended in saline or saline + 0.1% agar. C3H male and female mice (18-22 g, Charles River Laboratories, Wilmington, MA) were pharmacologically immunosuppressed for 7 days with methylprednisone, tacrolimus, and mycophenolate mofetil, and administered 40 g/L urea *ad libitum* in drinking water for 10 days to induce mild uremia, as previously described (14). Following immunosuppression and uremic induction, mice were administered 6 mg/kg flurofamide via IP injection (based on Ligon and Kenney 1990) (20), 1 h prior to IT and IP Ureaplasma challenge.

For IT challenge, mice were anesthetized with ketamine/xylazine (90/10 mg/kg), and 50 μL of bacterial suspension in saline + 0.1% agar was instilled using a 22G curved gavage needle, after which animals were held vertically for 10 min to allow distribution into the respiratory system. For IP challenge, 100 μL of bacterial suspension (10^7^-10^8^ organisms) was injected directly into the peritoneal cavity. Control groups consisted of saline vehicle with and without flurofamide treatment. After 24 h of infection, animals were sacrificed, blood collected for NH_3_ measurement with a point-of-care meter (Woodley Equipment Company LTD, WD5502 PocketChem BA Analyzer) and bacterial load quantification via qPCR, and lungs harvested for qPCR.

### Treatment of established hyperammonemia with flurofamide, *in vivo*.

Mice were immunosuppressed and urea-fed as described above, and infected IT and IP. Control groups consisted of saline vehicle with and without flurofamide treatment. Twenty-four h after infection, blood was collected from each animal via tail bleed and NH_3_ measured with a point-of-care meter. Infected animals exhibiting greater than 30 μmol/L NH_3_ were divided equally into 2 groups for flurofamide treatment and non-treatment. The treatment group was administered 6 mg/kg flurofamide via IP injection. Six h after treatment, animals were sacrificed, blood collected for NH_3_ measurement with a point-of-care meter and bacterial load quantified via qPCR, and lungs harvested for qPCR.

### qPCR assay.

DNA was purified from tissue and blood using a Maxwell RSC (Promega) per manufacturer instructions. Sample input was 200 μL and elution output 100 μL. qPCR for Ureaplasma was performed as previously described on a LightCycler 480 II (Roche Applied Science) (14, 23).

### Ethics statement.

This study was carried out in accordance with the recommendations in the Guide for the Care and Use of Laboratory Animals of the National Institutes of Health and was approved by Mayo Clinic Institutional Animal Care and Use Committee (protocol number: A5004-20). Mayo Clinic is AAALAC accredited (000717), registered with the USDA (41-R-0006), and has an Assurance with OLAW (A3291-01). Mice were housed in a biosafety level 2, specific-pathogen-free, AAALAC accredited facility, where sentinel mice are tested quarterly for murine pathogens; tested mice were negative for murine pathogens throughout the course of the study. Mice had unrestricted access to irradiated rodent food (LabDiet formula 5053) and water. The housing room was environmentally controlled (temperature 68–74°F, relative humidity 30–70%, 12:12-h light:dark cycle). All efforts were made to minimize suffering. Mice were monitored twice daily, and anesthetized mice monitored until awake. Animals were monitored for decreased activity, decreased body temperature, hunched stature, distress, and inability to eat and drink; if these findings were severe, animals were euthanized.
